# Supplementary material for: High drug-loaded microspheres enabled by controlled in-droplet precipitation promote functional recovery after spinal cord injury
Source: Nat Commun. 2022 Mar 10;13:1262. doi: 10.1038/s41467-022-28787-7 (PMC8913677; doi:10.1038/s41467-022-28787-7)
Supplement: Supplementary file 1 — Supplementary Information [file 41467_2022_28787_MOESM1_ESM.pdf]

# Supplementary Information

## High drug-loaded microspheres enabled by controlled in-droplet precipitation promote functional recovery after spinal cord injury

Wei Li[1,⊥], Jian Chen[2,⊥], Shujie Zhao[2], Tianhe Huang[3], Huiyan Ying[1], Claudia Trujillo[1], Giuseppina Molinaro[1], Zheng Zhou[2], Tao Jiang[2], Wei Liu[2], Linwei Li[2], Yuancheng Bai[3], Peng Quan[1,4], Yaping Ding[1], Jouni Hirvonen[1], Guoyong Yin[2*], Hélder A. Santos[1,5,6*], Jin Fan[2*] and Dongfei Liu[3,1,5*]

[1]Drug Research Program, Division of Pharmaceutical Chemistry and Technology, Faculty of Pharmacy, University of Helsinki, Helsinki 00014, Finland

[2]Department of Orthopaedics, The First Affiliated Hospital of Nanjing Medical University, Nanjing 210029, China

[3]State Key Laboratory of Natural Medicines, Department of Pharmaceutical Science, China Pharmaceutical University, Nanjing 210009, China

[4]Department of Pharmaceutical Science, School of Pharmacy, Shenyang Pharmaceutical University, Shenyang 110016, China

[5]Helsinki Institute of Life Science (HiLIFE), University of Helsinki, Helsinki 00014, Finland

[6]Department of Biomedical Engineering and W.J. Kolff Institute for Biomedical Engineering and Materials Science, University Medical Center Groningen / University of Groningen, Ant. Deusinglaan 1, 9713 AV Groningen, The Netherlands

[⊥]These authors contributed equally: Wei Li, Jian Chen.

Correspondence and requests for materials should be addressed to D.L. (e-mail: dongfei.liu@cpu.edu.cn) or to J.F. (e-mail: fanjin@njmu.edu.cn) or to H.A.S. (e-mail: h.a.santos@umcg.nl) or to G.Y. (email: guoyong_yin@sina.com)

## Supplementary Figures:

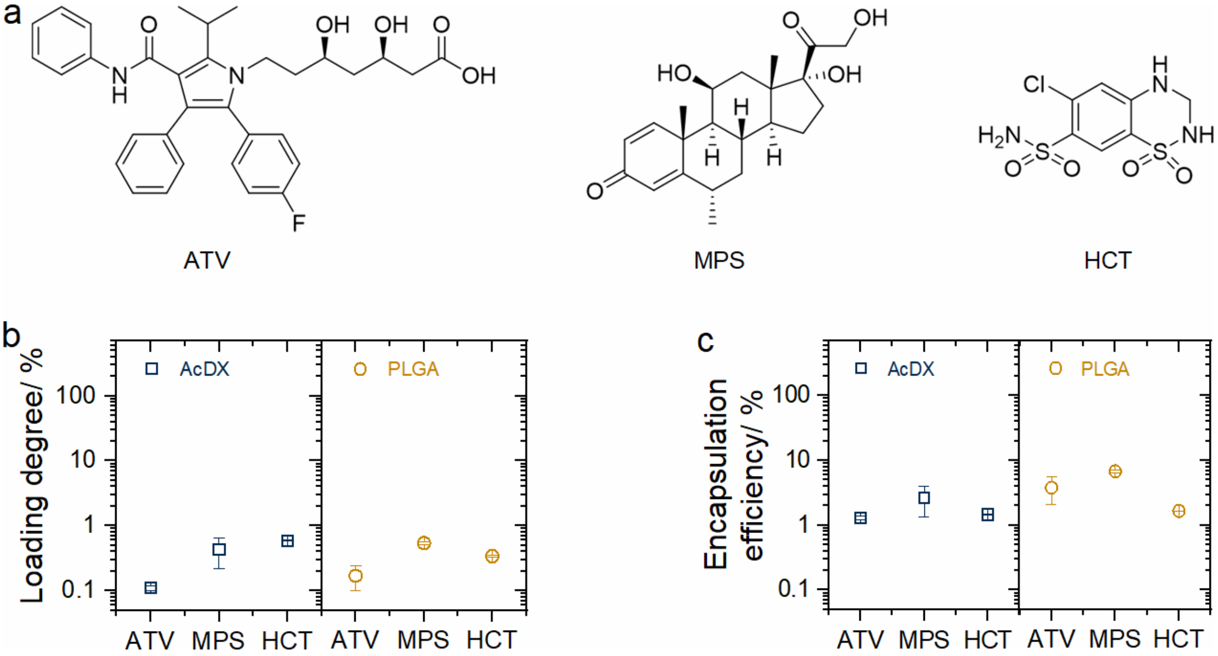


**Fig. S1**. (a) Molecular structures of drug molecules (ATV, MPS and HCT). (b and c) Experimental drug loading degree (b) and encapsulation efficiency (c) of drug-loaded polymer microspheres prepared using ethyl acetate as the solvent for the inner fluid.

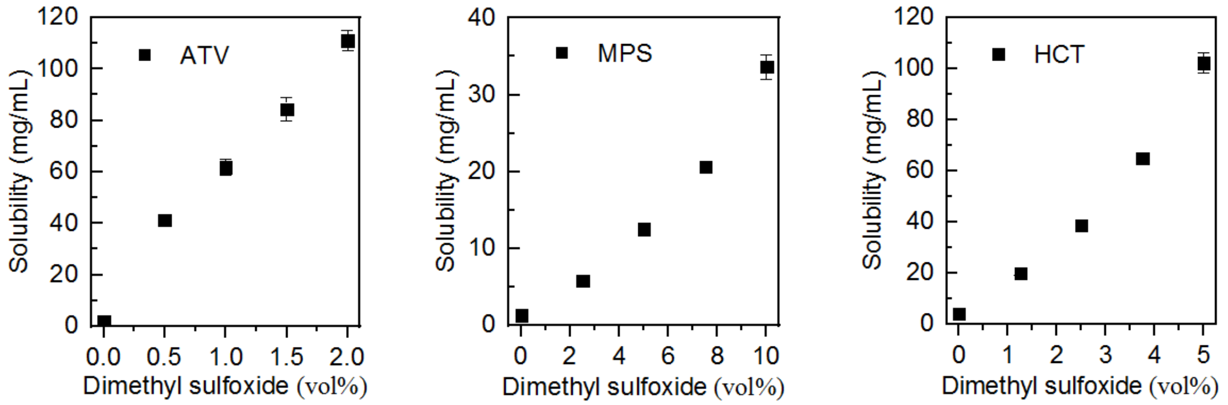


**Fig. S2.** The solubility of ATV, MPS and HCT in ethyl acetate with different ratio of dimethyl sulfoxide (*n* = 3). Data are presented as mean values ± SD.

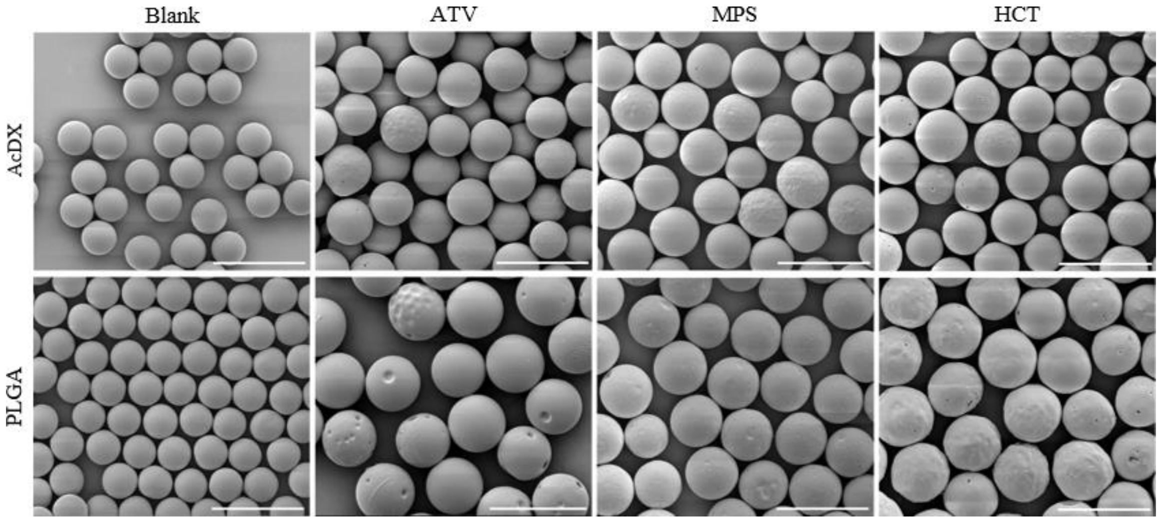


**Fig. S3.** Surface morphology of microspheres. Scanning electron microscope images of bare AcDX, ATV@AcDX, MPS@AcDX, HCT@AcDX, bare PLGA, ATV@PLGA, MPS@PLGA and HCT@PLGA microspheres prepared by microfluidic in-droplet precipitation method. Scale bars, 50 µm.

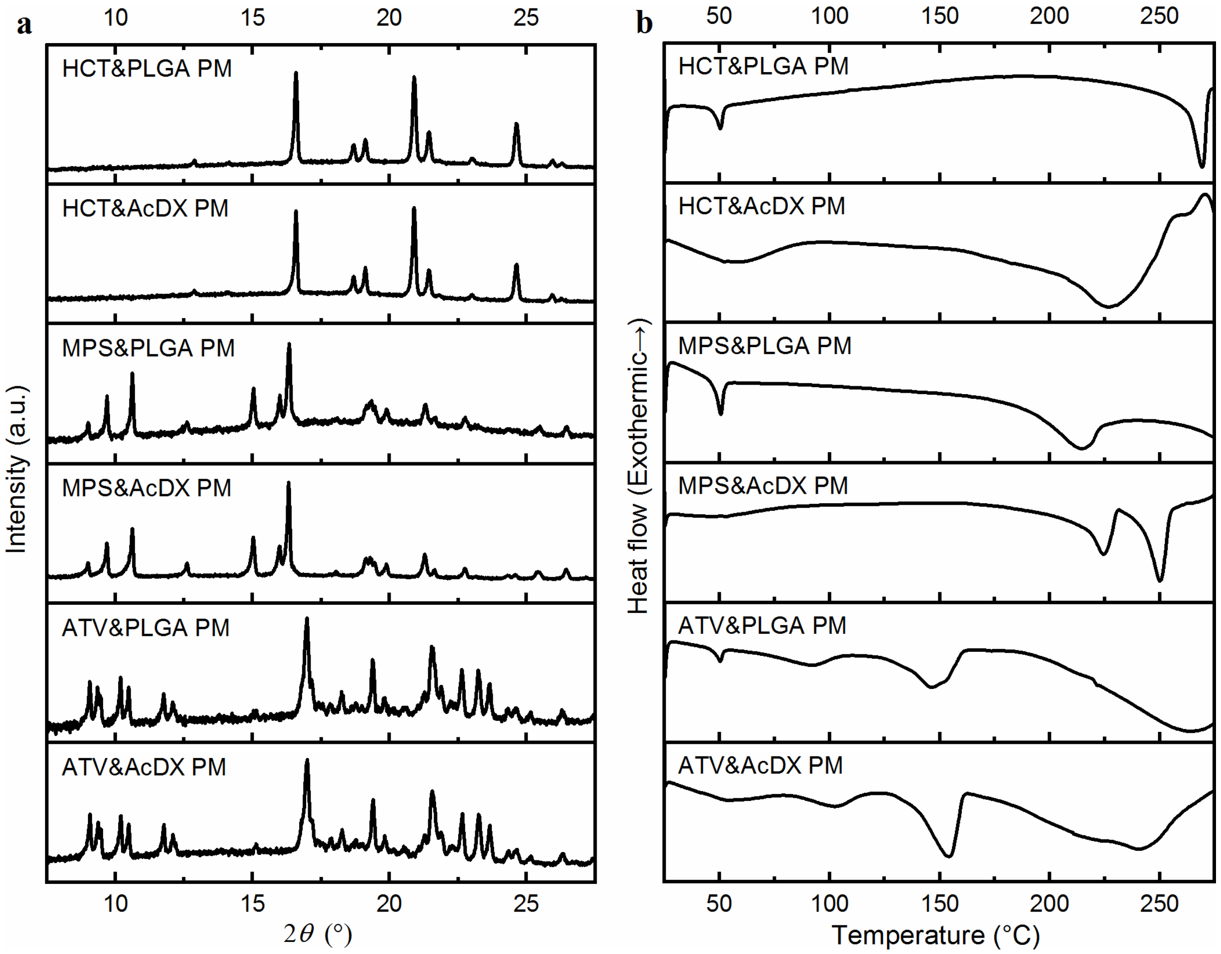


**Fig. S4**. Crystalline state of drugs in physical mixture (PM) of drug powder and bare microspheres. (**a**) X-ray powder diffractograms and (**b**) differential scanning calorimetry curves of ATV&AcDX PM, MPS&AcDX PM, HCT&AcDX PM, ATV&PLGA PM, MPS&PLGA PM and HCT&PLGA PM.

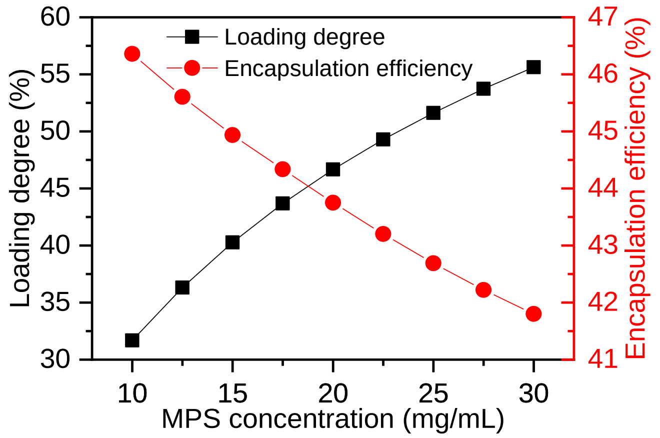


**Fig. S5.** Influence of MPS concentration in inner fluid on the loading degree and encapsulation efficiency of prepared microspheres. MPS is assumed to be freely soluble in the droplets.

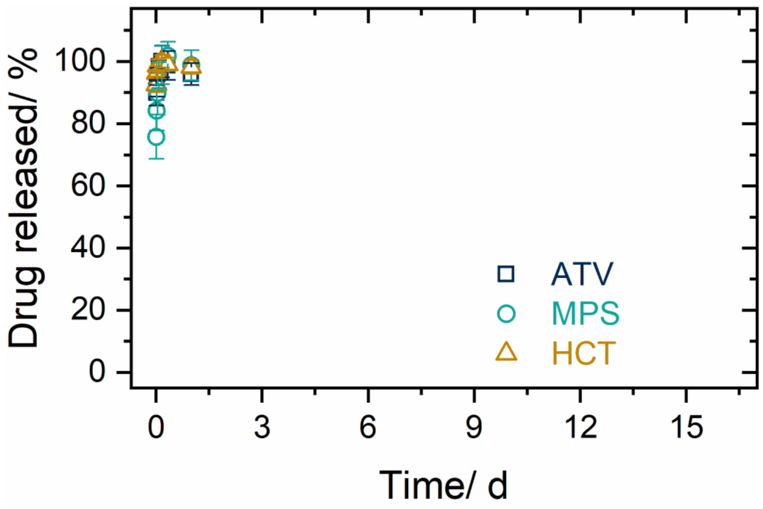


**Fig. S6.** Release profiles of ATV, MPS or HCT from each corresponding bare drug ($n$ = 3). Data are presented as mean values ± SD.

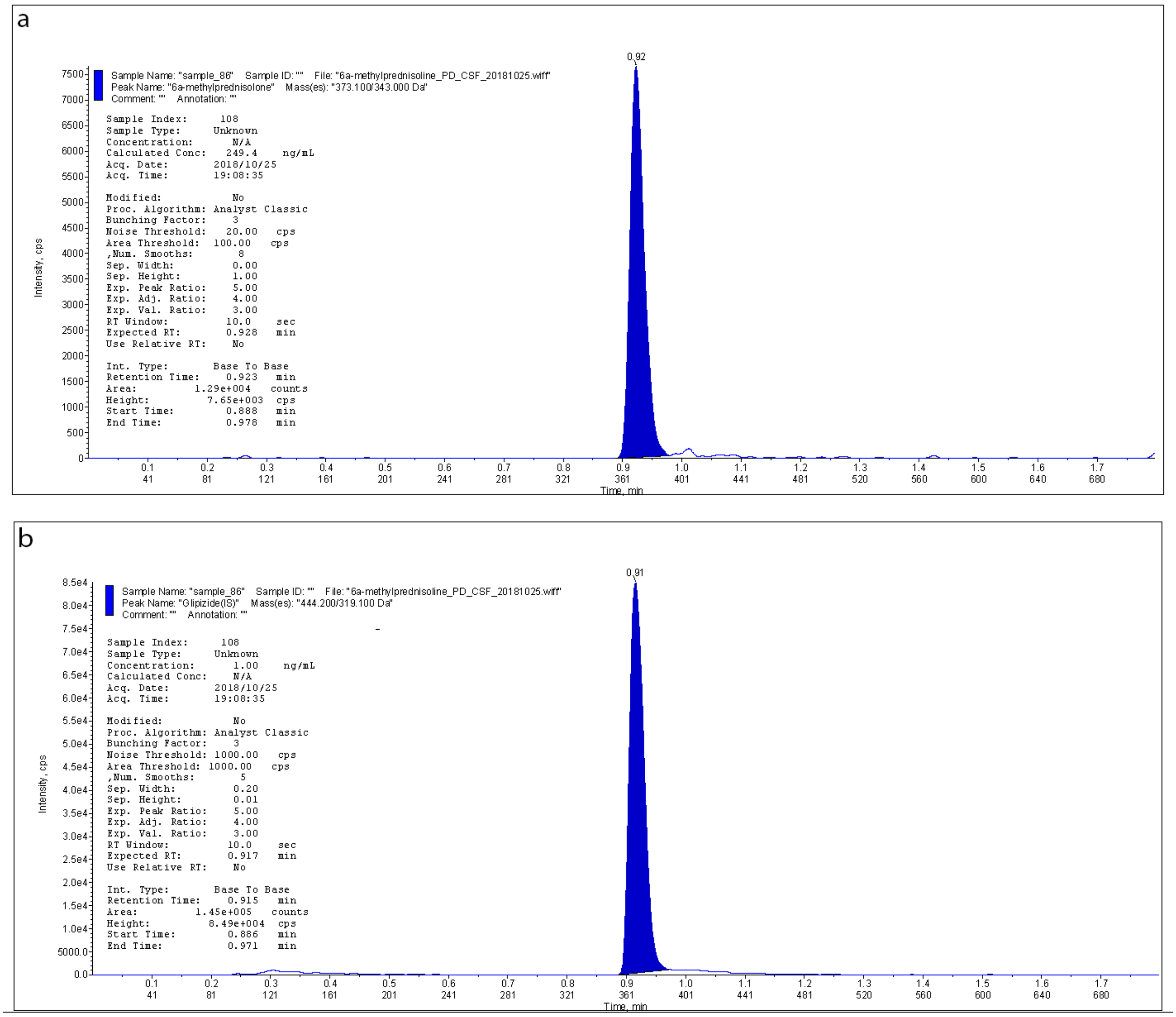


**Fig. S7.** LC–MS/MS chromatograms of MPS. (a) The representative LC–MS/MS chromatograms of MPS in a cerebrospinal fluid sample; (b) Chromatogram of the internal standard, glipizide.

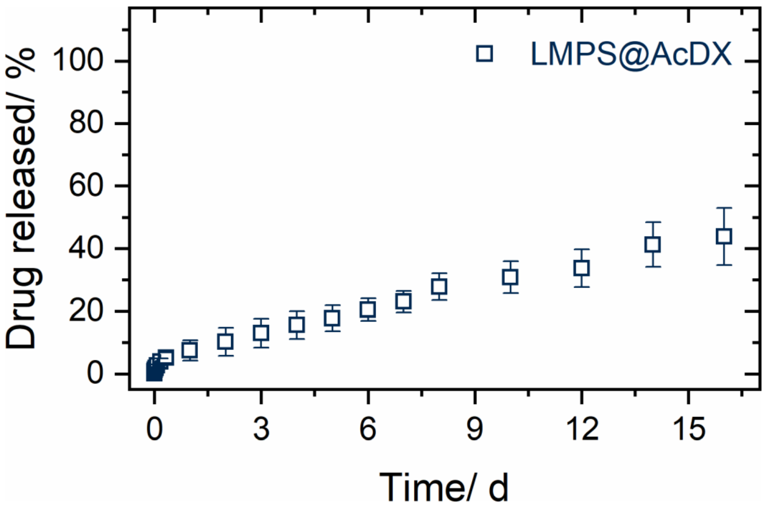


**Fig. S8.** MPS release profile from low MPS loaded AcDX microspheres ($n$ = 3). Data are presented as mean values ± SD.

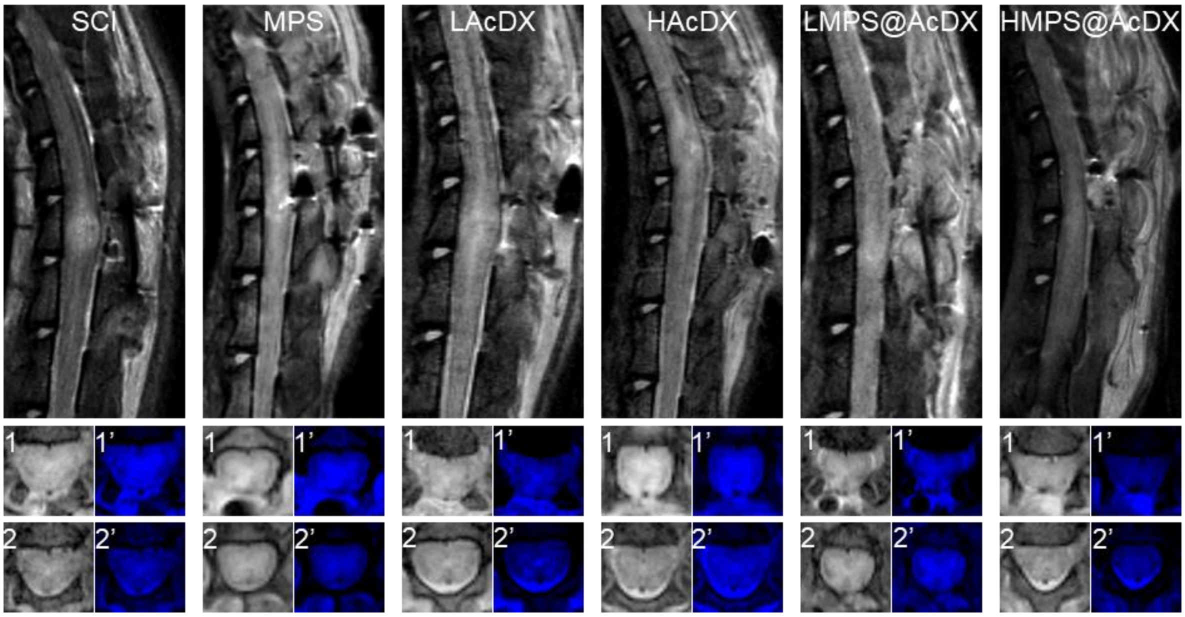


**Fig. S9.** Sagittal and axial spinal cord T2 weighted images at day-1 after injury. (1) & (1') are axial images in injury epicenter, (2) & (2') are axial images in far-injury area. (1') & (2') are axial color maps transformed from (1) and (2) normal axial images and the blue color represents the edema signal.

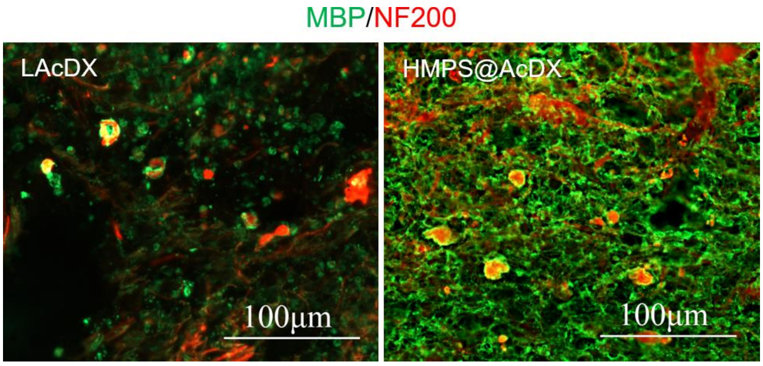


**Fig. S10**: Representative immunohistochemical staining of MBP (in green) and NF200 (in red) adjacent to the lesion area.

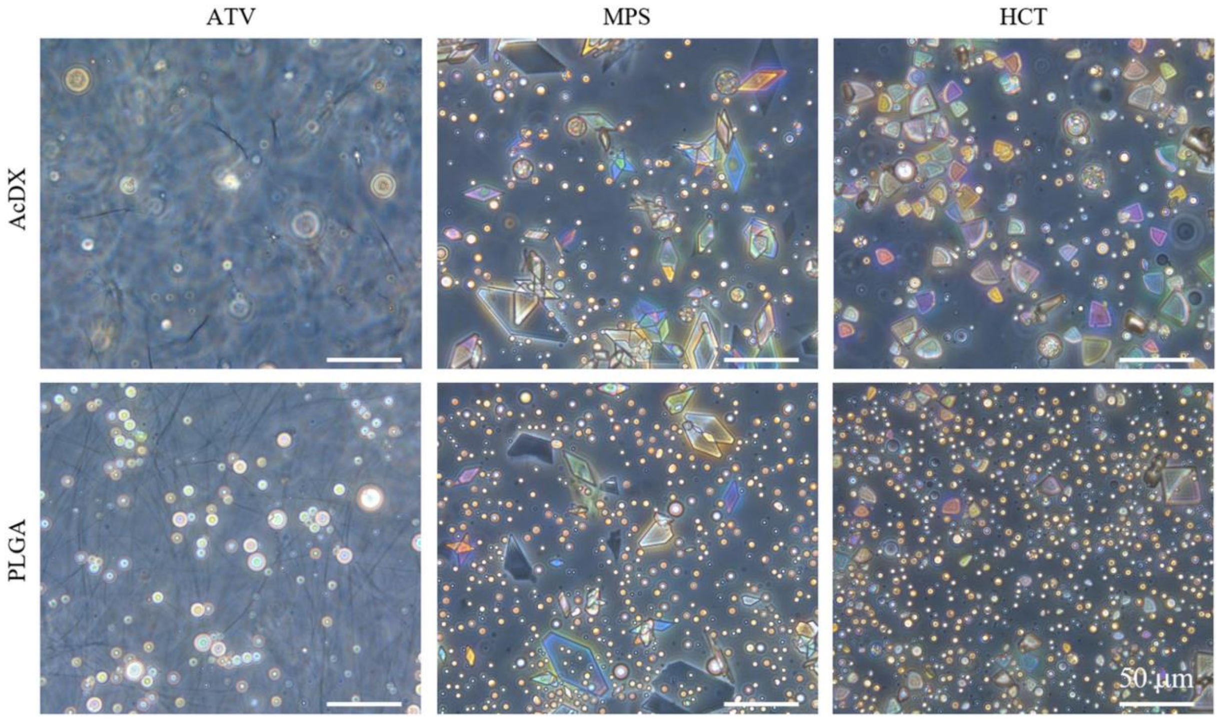


**Fig. S11.** Phase contrast microscope images of microspheres. ATV@AcDX, MPS@AcDX, HCT@AcDX, ATV@PLGA, MPS@PLGA and HCT@PLGA microspheres were prepared by magnetic stirring (bulk method). Scale bars, 50 µm.

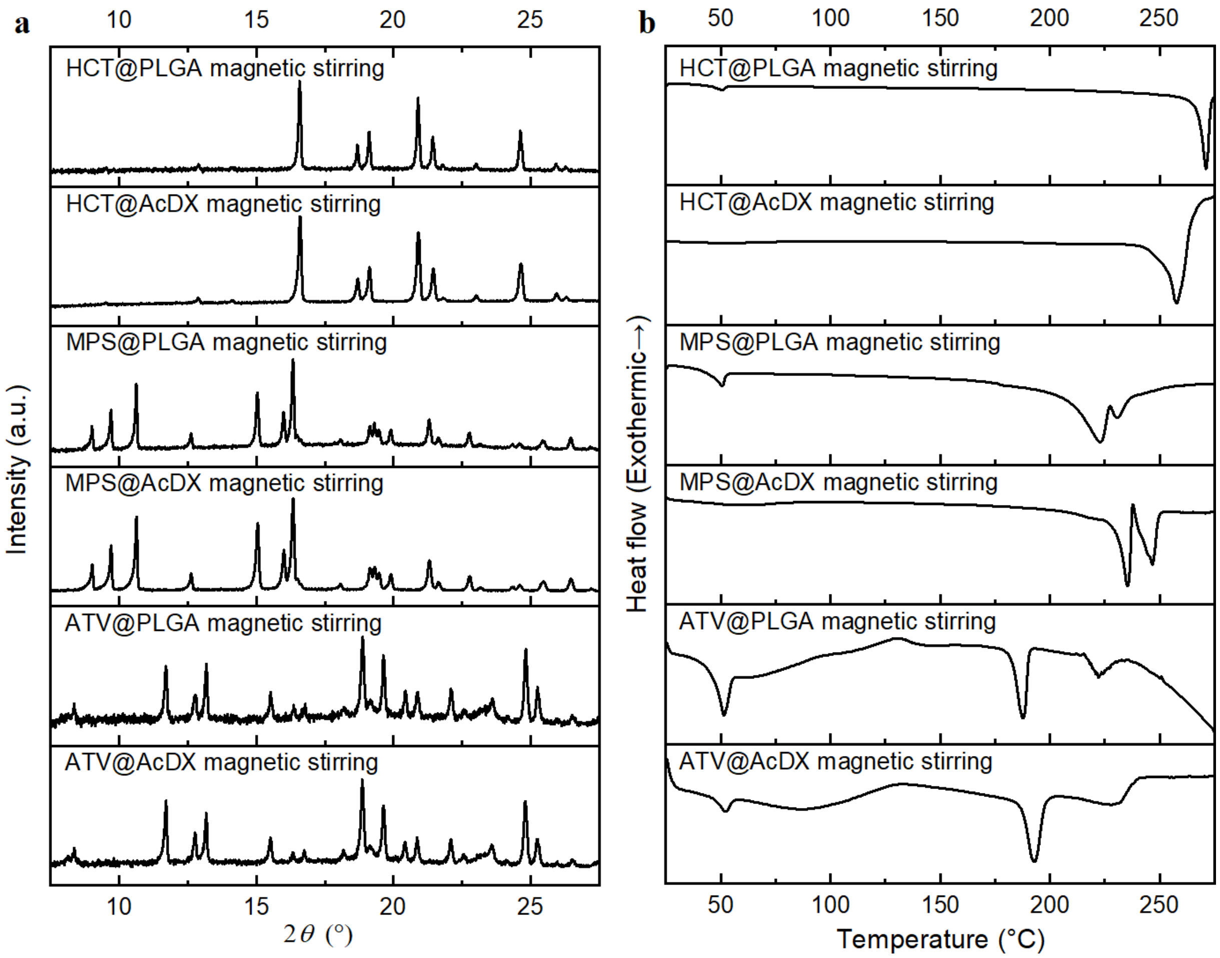


**Fig. S12.** Solid-state of drugs in microspheres. (a and b) X-ray powder diffractograms (a) and differential scanning calorimetry curves (b) of microspheres prepared by magnetic stirring (bulk method).

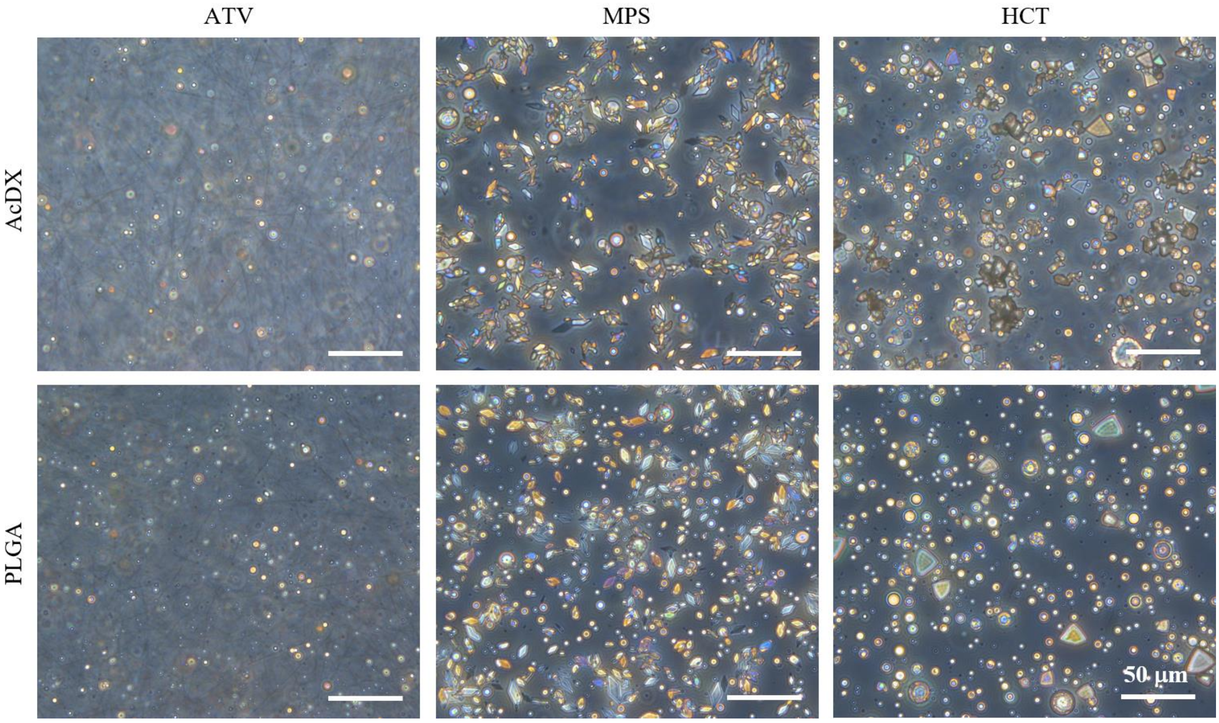


**Fig. S13**: Phase contrast microscope images of microspheres. ATV@AcDX, MPS@AcDX, HCT@AcDX, ATV@PLGA, MPS@PLGA and HCT@PLGA microspheres were prepared by homogenization (bulk method) using ethyl acetate as the primary solvent. Scale bars, 50 μm.

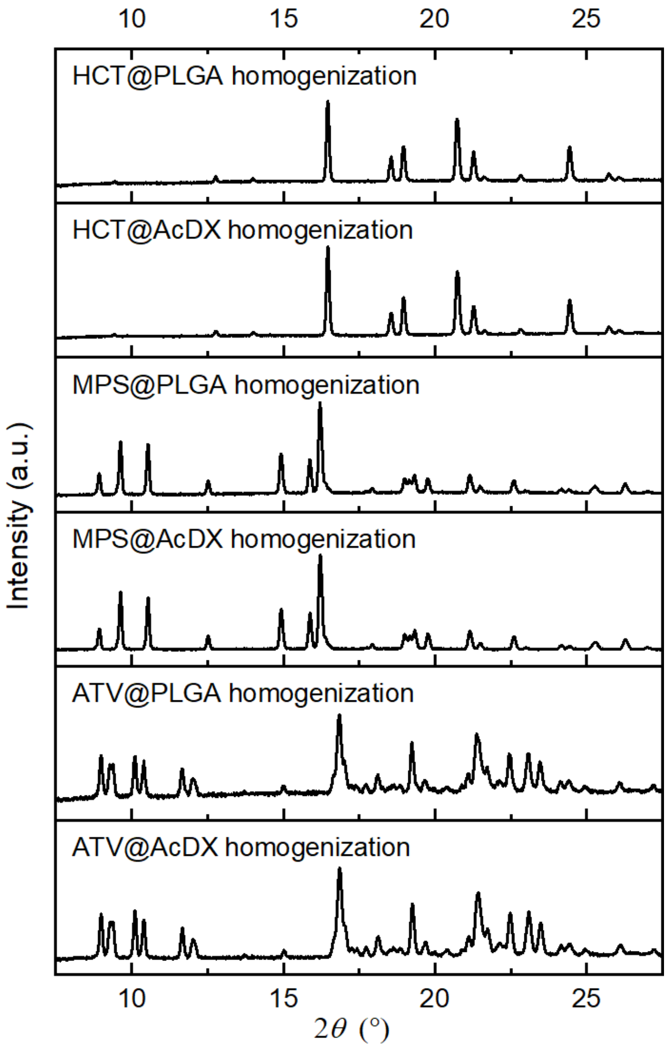


**Fig. S14.** X-ray powder diffractograms of microspheres prepared by homogenization (bulk method). Ethyl acetate was used as the primary solvent for preparing the microspheres.

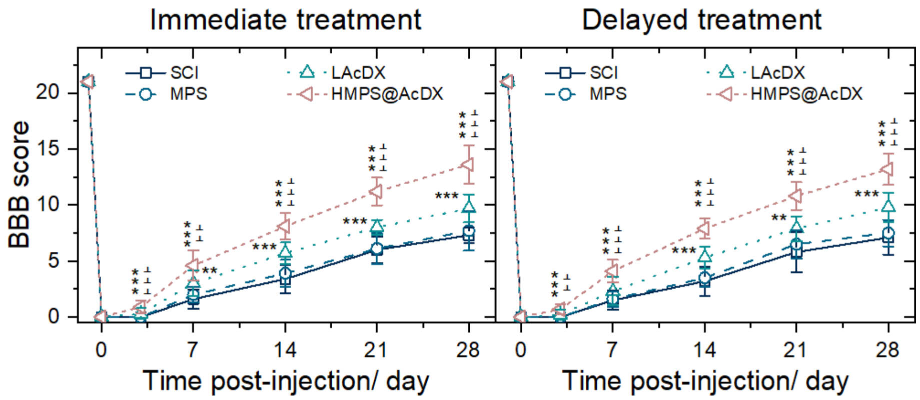


**Fig. S15**: The rats were functionally graded up to 28 days post-injury by BBB grading scale, *n* = 10 rats per group. The intervention groups were compared with the SCI group ($^{*}$); HMPS@AcDX group was compared with LAcDX group ($^{\perp}$); statistical significance was analyzed using one-way ANOVA followed by Fisher's post-hoc test. Delayed treatment group was compared with immediate treatment group ($^{\#}$); statistical significance was analyzed using pair-sample *t*-test. $^{**}$, $^{\perp\perp}$ $P < 0.01$, $^{***}$, $^{\perp\perp\perp}$ $P < 0.001$. Exact *P* values are given in the Source Data file. For both immediate treatment and delayed treatment, throughout the entire assessment period, HMPS@AcDX recovered motor function significantly faster than the other groups including LAcDX. In addition, for HMPS@AcDX, there was no significant difference in the BBB score between immediate treatment and delayed treatment. Data are presented as mean values ± SD.

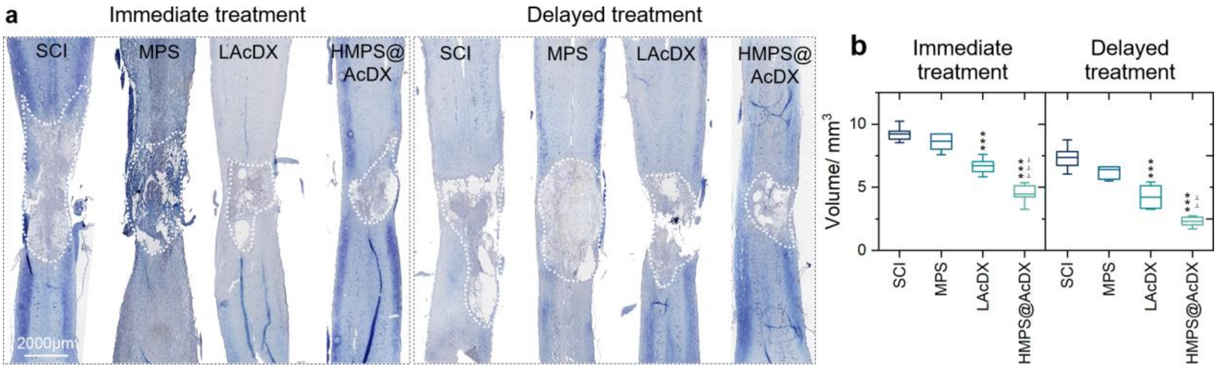


**Fig. S16**: (**a**) Sagittal spinal cord sections stained with Nissl display the injury area of spinal cord at day-28 post trauma. (**b**) Comparison of the lesion volume after treatment ($n$ = 5 rats per group). Box plots show the minimum value, the first quartile, the median, the third quartile, and the maximum value. The intervention groups were compared with the SCI group ($^*$); HMPS@AcDX group was compared with LAcDX group ($^\perp$); statistical significance was analyzed using one-way ANOVA followed by Fisher's post-hoc test. Delayed treatment group was compared with immediate treatment group ($^\#$); statistical significance was analyzed using pair-sample $t$-test. $^{\perp\perp}$ $P < 0.01$, $^{***}$, $^{\perp\perp\perp}$ $P < 0.001$. Exact $P$ values are given in the Source Data file. Four weeks after injury, the lesion volume in the groups treated by LAcDX ($P < 0.001$) and HMPS@AcDX ($P < 0.001$) was notably smaller than that of the SCI group for both immediate treatment and delayed treatment. In comparison with LAcDX, HMPS@AcDX significantly reduced the loss of post-traumatic spinal cord tissue ($P < 0.001$ for immediate treatment and $P < 0.01$ for delayed treatment). In addition, for HMPS@AcDX, there was no significant difference in the lesion volume between immediate treatment and delayed treatment.

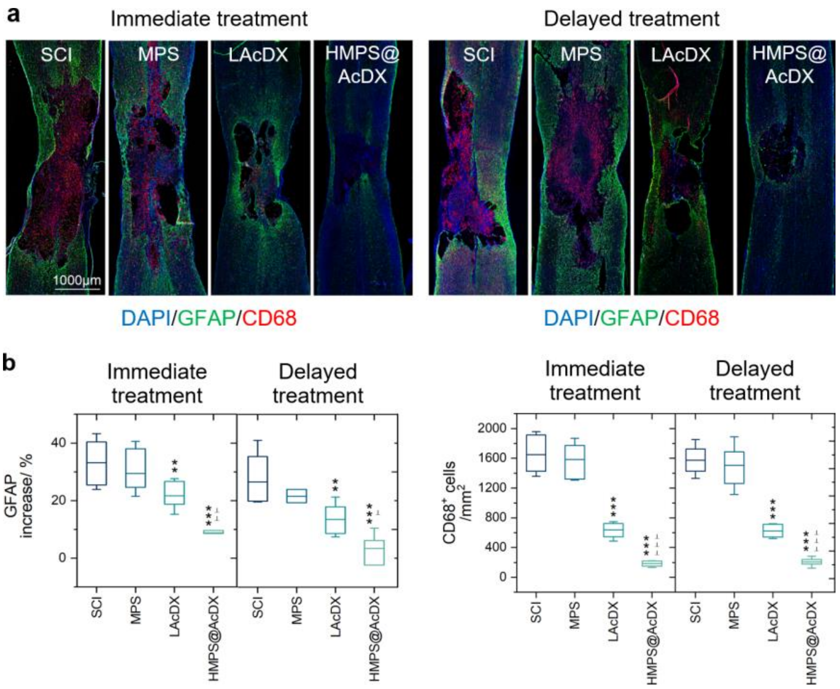


**Fig. S17**: (**a**) Representative immunohistochemical staining of GFAP (in green) and CD68 (in red) in longitudinal sections of injured spinal cord at day-28 post injury. The nuclei of all cells were stained with DAPI (in blue). (**b**) Semi-quantification of GFAP intensity and density of microglia in the injured spinal cord. For GFAP intensity, the data are plotted as the relative ratio of the immunoreactivity near the injury site compared with that in distant area. ($n$ = 6 per group). Box plots show the minimum value, the first quartile, the median, the third quartile, and the maximum value. The intervention groups were compared with the SCI group ($^{*}$); HMPS@AcDX group was compared with LAcDX group ($^{\perp}$); statistical significance was analyzed using one-way ANOVA followed by Fisher's post-hoc test. Delayed treatment group was compared with immediate treatment group ($^{\#}$); statistical significance was analyzed using pair-sample $t$-test. $^{\perp}$ $P < 0.05$, $^{**}$, $^{\perp\perp}$ $P < 0.01$, $^{***}$, $^{\perp\perp\perp}$ $P < 0.001$. Exact $P$ values are given in the Source Data file. As compared to SCI group, the treatment with LAcDX ($P < 0.01$) and HMPS@AcDX ($P < 0.001$) significantly inhibited the increase of GFAP immunoreactivity for both immediate treatment and delayed treatment. In comparison with LAcDX, HMPS@AcDX significantly inhibited the increase of GFAP immunoreactivity ($P < 0.01$ for immediate treatment and $P < 0.05$ for delayed treatment). In addition, for HMPS@AcDX, there was no significant difference in the GFAP immunoreactivity between immediate treatment and delayed treatment. The density of CD68-positive microglia in the lesion area for LAcDX was significantly smaller than that in SCI group ($P < 0.001$ for both immediate treatment and delayed treatment). HMPS@AcDX further significantly reduced the number of microglia in the traumatic lesion area compared to LAcDX group ($P < 0.001$ for both immediate treatment and

delayed treatment). In addition, for HMPS@AcDX, there was no significant difference in the density of CD68-positive microglia between immediate treatment and delayed treatment.

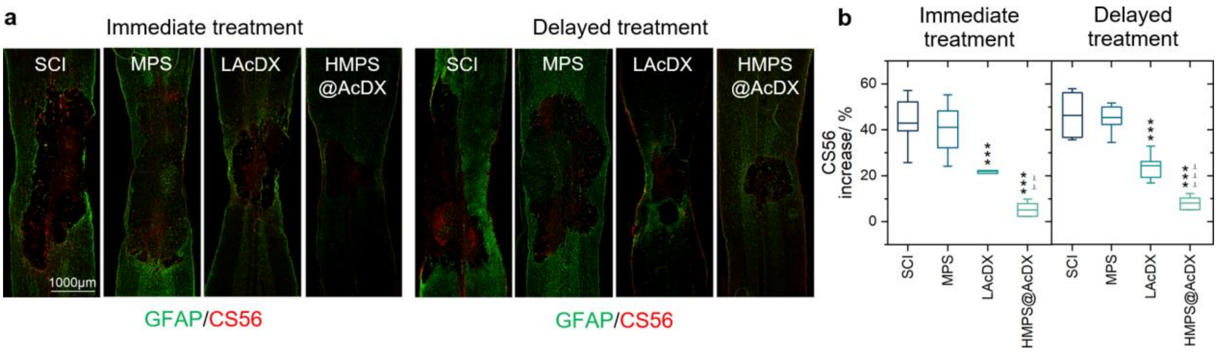


**Fig. S18**: (**a**) The deposition of chondroitin sulfate proteoglycans (CSPGs) determined by CS56 antibody (in red) at day-28 after injury. (**b**) Semi-quantification of CS56 intensity increase in the traumatic lesion area. The data are plotted as the relative ratio of the immunoreactivity near the injury site compared with that in the distant area. (*n* = 6 per group). Box plots show the minimum value, the first quartile, the median, the third quartile, and the maximum value. The intervention groups were compared with the SCI group ($^{*}$); HMPS@AcDX group was compared with LAcDX group ($^{\perp}$); statistical significance was analyzed using one-way ANOVA followed by Fisher's post-hoc test. Delayed treatment group was compared with immediate treatment group ($^{\#}$); statistical significance was analyzed using pair-sample *t*-test. $^{\perp\perp}$ $P < 0.01$, $^{***}$, $^{\perp\perp\perp}$ $P < 0.001$. Exact *P* values are given in the Source Data file. The CSPG level for LAcDX was significantly smaller than that in SCI group ($P < 0.001$ for both immediate treatment and delayed treatment). HMPS@AcDX further significantly reduced the CSPG level compared to LAcDX group ($P < 0.01$ for immediate treatment and $P < 0.001$ for delayed treatment). In addition, for HMPS@AcDX, there was no significant difference in the CSPG level between immediate treatment and delayed treatment.

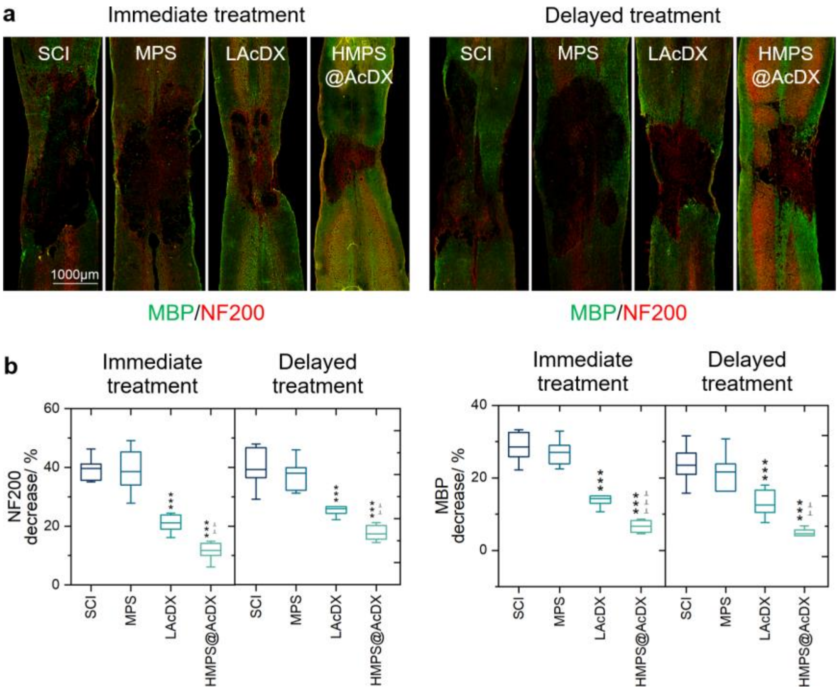


**Fig. S19**: (**a**) Immunohistochemical staining of MBP (in green) and NF200 (in red) in injured spinal cord at day-28 post trauma. (**b**) Semi-quantification of NF200 and MBP intensity after spinal cord injury therapy. The data are plotted as the relative ratio of the immunoreactivity near the injury site compared with that in distant area. ($n$ = 6 per group). Box plots show the minimum value, the first quartile, the median, the third quartile, and the maximum value. The intervention groups were compared with the SCI group ($^{*}$); HMPS@AcDX group was compared with LAcDX group ($^{\perp}$); statistical significance was analyzed using one-way ANOVA followed by Fisher's post-hoc test. Delayed treatment group was compared with immediate treatment group ($^{\#}$); statistical significance was analyzed using pair-sample $t$-test. $^{\perp\perp}$ $P < 0.01$, $^{***}$, $^{\perp\perp\perp}$ $P < 0.001$. Exact $P$ values are given in the Source Data file. For both immediate treatment and delayed treatment, in comparison with SCI group, a significant decrease in NF200 intensity was observed for LAcDX ($P < 0.001$) and HMPS@AcDX ($P < 0.001$) groups. HMPS@AcDX further significantly reduced the NF200 intensity compared to LAcDX group ($P < 0.01$ for both immediate treatment and delayed treatment). In addition, for HMPS@AcDX, there was no significant difference in the NF200 intensity between immediate treatment and delayed treatment. In comparison with SCI group, a significant decrease in MBP intensity was observed for LAcDX ($P < 0.001$ for both immediate treatment and delayed treatment) and HMPS@AcDX ($P < 0.001$ for both immediate treatment and delayed treatment) groups. HMPS@AcDX further significantly reduced the MBP intensity compared to LAcDX group ($P < 0.001$ for immediate treatment and $P < 0.01$ for delayed treatment). In addition, for

HMPS@AcDX, there was no significant difference in the MBP intensity between immediate treatment and delayed treatment.

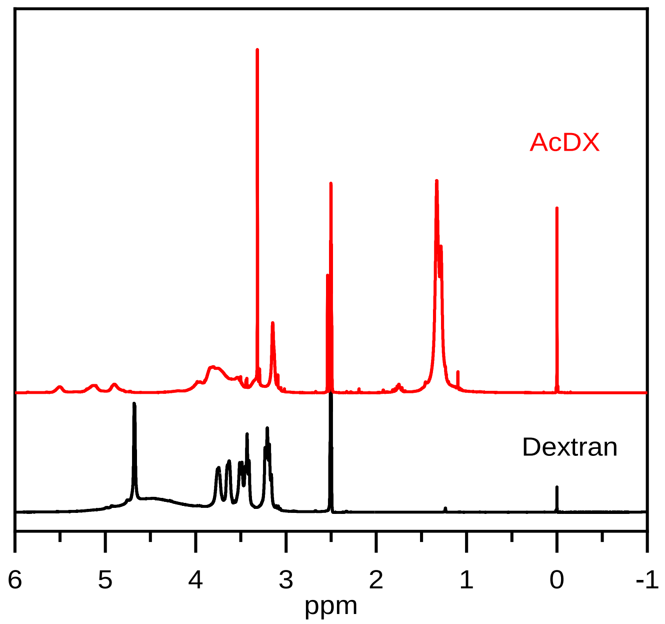


**Fig. S20.** Nuclear magnetic resonance spectra of the dextran and the synthesized AcDX.

**Supplementary Tables:**

**Table S1.** Simulated and experimental loading degree, encapsulation efficiency and particle size of HAcDX@MPS microspheres. Data are presented as mean values ± SD.

| Method | Loading degree (wt %) | Encapsulation efficiency (%) | Particle size (μm) |
|---|---|---|---|
| Simulation | 62.9 | 56.6 | 24.4 |
| Experiment | 63.1 ± 2.3 | 57.2 ± 5.5 | 25.8 ± 1.1 |

Note: Microspheres were prepared using inner fluid consisting of 10 mg/mL AcDX and 30 mg/mL MPS in a mixture of dimethyl sulfoxide and ethyl acetate (1:9, v/v).

**Table S2.** The mass of samples and volume of release media for drug release studies.

| Sample name | Mass of sample (mg) | Volume of release media (mL) |
|---|---|---|
| ATV | 1.7 | 50 |
| ATV@AcDX | 3.7 | 50 |
| ATV@PLGA | 3.0 | 50 |
| MPS | 1.2 | 50 |
| MPS@AcDX | 1.8 | 50 |
| MPS@PLGA | 5.0 | 50 |
| HCT | 2.7 | 50 |
| HCT@AcDX | 12.7 | 50 |
| HCT@PLGA | 5.7 | 50 |

**Table S3.** Parameters and their values used for the simulation of droplet solidification process.

| Parameter | Value |
|---|---|
| Initial concentration of AcDX | 10 (g $L^{-1}$) |
| Diffusion coefficient of AcDX in droplet | Infinitesimal ($m^2$ $s^{-1}$) |
| Diffusion coefficient of water in droplet | $6.666\times10^{-(9+4\times phi\_p)}$ ($m^2$ $s^{-1}$) |
| Diffusion coefficient of ethyl acetate in droplet | $2.693\times10^{-(9+4\times phi\_p)}$ ($m^2$ $s^{-1}$) |
| Diffusion coefficient of ethyl acetate in water | $0.952\times10^{-9}$ ($m^2$ $s^{-1}$) |
| Diffusion coefficient of dimethyl sulfoxide in water | $1.008\times10^{-9}$ ($m^2$ $s^{-1}$) |
| Convective mass transfer coefficient of water | 0.002469 (m $s^{-1}$) |
| Molar mass of AcDX | 10000 (g $mol^{-1}$) |
| Molar mass of water | 18 (g $mol^{-1}$) |
| Molar mass of ethyl acetate | 88.11 (g $mol^{-1}$) |
| Molar mass of MPS | 374.47 (g $mol^{-1}$) |
| Density of ethyl acetate | 0.902 (g $cm^{-3}$) |
| Density of water | 1 (g $cm^{-3}$) |
| Density of MPS | 1.3 (g $cm^{-3}$) |
| Diffusion coefficient of MPS in ethyl acetate | $1.284\times10^{-(9+4\times phi\_p)}$ ($m^2$ $s^{-1}$) |
| Diffusion coefficient of MPS in dimethyl sulfoxide | $0.270\times10^{-(9+4\times phi\_p)}$ ($m^2$ $s^{-1}$) |
| Diffusion coefficient of MPS in water | $0.497\times10^{-9}$ ($m^2$ $s^{-1}$) |
