# Supplementary material for: High drug-loaded microspheres enabled by controlled in-droplet precipitation promote functional recovery after spinal cord injury
Source: Nat Commun. 2022 Mar 10;13:1262. doi: 10.1038/s41467-022-28787-7 (PMC8913677; doi:10.1038/s41467-022-28787-7)
Supplement: Supplementary file 3 — Description of Additional Supplementary Files [file 41467_2022_28787_MOESM3_ESM.docx]

**Description of Additional Supplementary Files**

File Name: Supplementary Movie 1

Description: Droplets generated by flow-focusing microfluidic device pass through the spiral-shaped capillary tube under ultraviolet irradiation.
